# Micro and Nanoplastics and Obstetric Outcomes in Humans and Animals: A Systematic Review

**DOI:** 10.3390/ijerph23050672

**Published:** 2026-05-19

**Authors:** Blanca Novillo-Del Álamo, Alicia Martínez-Varea, Imelda Ontoria-Oviedo, Alba Ruiz-Gaitán, Charlotte Cosemans, Michelle Plusquin, Beatriz Marcos-Puig

**Affiliations:** 1 Department of Obstetrics and Gynaecology, La Fe University and Polytechnic Hospital, Avenida Fernando Abril Martorell 106, 46026 Valencia, Spainmarcos_bea@gva.es (B.M.-P.); 2 Departamento de Medicina y Cirugía, Facultad de Ciencias de la Salud, Universidad Cardenal Herrera-CEU, CEU Universities, C/Santiago Ramón y Cajal S/N, 46115 Valencia, Spain; 3 La Fe Medical Research Institute, Avenida Fernando Abril Martorell 106, Torre A, 46026 Valencia, Spain; imelda_ontoria@iislafe.es (I.O.-O.); alba_ruiz@externos.iislafe.es (A.R.-G.); 4 Department of Pediatrics, Obstetrics and Gynecology, Faculty of Medicine, University of Valencia, 12006 Valencia, Spain; 5 Department of Medical Microbiology, La Fe University and Polytechnic Hospital, Avenida Fernando Abril Martorell 106, 46026 Valencia, Spain; 6 Centre for Environmental Sciences (CMK), Hasselt University, 3590 Diepenbeek, Belgium; charlotte.cosemans@uhasselt.be (C.C.); michelle.plusquin@uhasselt.be (M.P.)

**Keywords:** plastic, placenta, toxicity, oxidative stress, intrauterine growth restriction, preterm birth

## Abstract

**Highlights:**

**Public health relevance—How does this work relate to a public health issue?**

- Micro- and nanoplastics exposure is an emerging environmental concern with potential effects on maternal and fetal health.
- This systematic review synthesizes current evidence on the possible association between micro- and nanoplastics and adverse obstetric outcomes, including preterm birth, low birth weight, intrauterine growth restriction and miscarriage.

**Public health significance—Why is this work of significance to public health?**

- Exposure to micro- and nanoplastics is widespread in the general population.
- Their potential association with impaired fetal growth, prematurity and miscarriage represents a significant public health issue with implications for maternal–fetal health and long-term population outcomes.

**Public health implications—What are the key implications or messages for practitioners, policy makers and/or researchers in public health?**

- Findings support that healthcare professionals should consider micro- and nanoplastics as potential risk factors during pregnancy.
- These and forthcoming high-quality studies will shed light on exposure routes, biological mechanisms, and clinical consequences that will serve as a foundation for future preventive policy strategies.

**Abstract:**

**Background**: Micro- and nano-plastics (MNPs) are pervasive environmental contaminants that accumulate in various tissues, including the placenta. Experimental and clinical studies suggest potential cytotoxic, oxidative, and inflammatory effects that may lead to placental dysfunction and adverse obstetric outcomes. However, high-quality evidence on the clinical relevance of MNPs exposure during pregnancy remains scarce, underscoring the need for systematic evaluation of their impact on maternal and fetal health. **Methods**: The databases PubMed, ScienceDirect, CENTRAL, Embase, MDPI and Google Scholar were searched for studies published up to September 2025 investigating the relationship between MNPs and obstetric outcomes. **Results**: Twelve studies were included in this review, with half employing an observational design in human subjects and the other half using experimental studies in murine models. Although the available evidence is limited, there are studies reporting the association between MNPs exposure and premature birth, low birth weight, intrauterine growth restriction, and miscarriage. The most prevalent polymer detected was polyethylene, and the most commonly used MNPs detection techniques were Raman microspectroscopy, digital microscopy, Fourier Transform Infrared, and Pyrolysis gas chromatography-mass spectrometry. **Conclusions**: This systematic review summarizes current limited insights on the potential effects of MNPs on obstetric outcomes, highlighting possible associations with low gestational age, low birth weight, intrauterine growth restriction, and miscarriage. Findings do not allow causal inference due to heterogeneity in study design, exposure assessment, contamination control, and analytical methodologies.

## 1. Introduction

Microplastics are plastic particles measuring 5 mm or less in size, whereas nanoplastics are generally defined as particles with a size of 1 μm or smaller [1]. Collectively, micro- and nano-plastics (MNPs) (≤5 mm) are ubiquitous [1,2]. The main exposure routes include ingestion (via drinking water and food), inhalation, and dermal contact [2,3,4,5,6,7]. Tap water has been compared with bottled water, with the latter found to contain significantly higher levels of MNPs, suggesting that drinking tap water could reduce our intake of MNPs [5].

MNPs have been shown to accumulate in biological tissues [2,3,4,5,6,7,8,9]. The lung is the organ where the highest detection of MNPs has been recorded [6]. A greater accumulation has also been reported in females compared to males [10]. It has been hypothesized that their presence may correlate with ultrastructural alteration in specific intracellular organelles—primarily mitochondria and endoplasmic reticulum—potentially leading to multiorgan effects through cytotoxicity, oxidative stress, inflammatory response, genotoxicity, hepatotoxicity, neurotoxicity, renal toxicity, and even carcinogenicity, ultimately resulting in clinical disorders [11,12,13].

The detection of MNPs in mammalian placentas, including human [13,14,15], as well as meconium, amniotic fluid, cord blood, and breast milk, has been demonstrated in both experimental and clinical settings [13,15,16,17,18]. These findings suggest a possible association with placental dysfunction [19,20]. Furthermore, a study investigating the effects of MNPs on human endometrial stromal cells found that smaller plastics exhibited a higher propensity for cellular uptake, with significant morphological alterations and cell death observed at concentrations above 100 μg/mL after 24 h of exposure [21]. MNPs exposure has also been linked to female infertility through apoptosis of ovarian granulosa cells [22,23]. However, findings regarding the effect on offspring remain inconsistent, with studies reporting contradictory results [23,24,25,26,27]. While the detection of MNPs in tissues demonstrates exposure and translocation, it does not necessarily imply biological or clinical relevance. The distinction between presence and pathogenic effect remains a major gap in the current evidence.

The most comprehensive systematic review to date regarding systemic symptoms related to MNPs, encompassing 133 studies, reported harmful effects in 117 studies, whereas 16 studies found no significant impact on human health [12]. Associations between MNPs exposure and adverse reproductive and obstetric outcomes—including growth restriction, stillbirth, preterm birth (PTB), and infertility—have been suggested [28,29], as well as an influence on fetal health [30]. Several studies have addressed the detection of MNPs in the placenta, as well as hypotheses regarding their potential adverse effects and underlying mechanisms of action. Nevertheless, high-quality studies providing robust evidence on the actual clinical implications of MNPs exposure in obstetric outcomes remain notably limited [17,28,31,32,33].

MNPs are recognized as emerging environmental pollutants [34]. Historically, research on pollution has focused primarily on gaseous components and their harmful health effects [35,36,37]. However, recent studies have begun to consider MNPs as an additional airborne contaminant. For instance, a Canadian cross-sectional study analyzed not only carbon, graphite, and lead oxide as pollutants in human placenta, but also MNPs [14]. A prospective case–control study conducted in the United Kingdom found no association between ambient air pollution and stillbirth rates. However, it suggested that periconceptual exposure to NO_2_ may represent a risk factor [34]. Nevertheless, they did not take into account MNPs [34].

The authors therefore consider this systematic review essential for synthesizing existing evidence on the effects of MNPs exposure on obstetric outcomes in animals and humans, moving beyond detection and mechanistic hypotheses toward a clearer understanding of their true clinical significance.

## 2. Materials and Methods

This systematic review was conducted in accordance with the PRISMA guidelines ([38] File S1). 

### 2.1. Literature Search

The literature search was conducted in accordance with the PICO framework:

Population: Pregnant human individuals in any setting, with documented or inferred exposure to MNPs, as well as animal models exposed to MNPs to investigate obstetric outcomes.

Intervention/Exposure: Exposure to MNPs through any route, including ingestion, inhalation, dermal contact, environmental exposure, or experimental administration.

Comparator: None or lower exposure to MNPs.

Outcomes: Miscarriage, stillbirth, fetal growth, birth weight (BW), and gestational age (GA) at delivery.

A comprehensive literature search was independently conducted by two reviewers across the following databases: PubMed, Embase, ScienceDirect, CENTRAL, MDPI, and Google Scholar, covering all records available as of October 2025.

For PubMed and Embase, controlled vocabulary terms were combined with free-text keywords. Medical Subject Headings (MeSH) and Emtree terms included “plastics”, “environmental pollution”, “environmental exposure”, “particulate matter”, “placenta”, “pregnancy”, “pregnancy outcome”, “fetal development”, and “birth weight”. These were complemented with free-text terms such as “microplastics”, “nanoplastics”, “MNPs”, “preterm birth”, “stillbirth”, and “miscarriage”.

On ScienceDirect, CENTRAL, and MDPI, where controlled vocabularies are limited, searches were conducted using combinations of keywords in titles, abstracts, and full text. Google Scholar was used as a supplementary source to identify additional relevant studies, screening the first 200 results in relevance order.

Search strategies were tailored to the specific syntax of each database and combined using Boolean operators, with exposure-related terms linked by “OR” and outcome-related terms linked by “OR”, and both concept blocks combined using “AND”.

All retrieved references were imported into ZOTERO (version 6.0.30) for reference management and duplicate removal. Studies published up to October 2025 were considered eligible for initial screening.

This systematic review was registered in PROSPERO (CRD420251233886).

### 2.2. Eligibility Criteria

The inclusion criteria were the following: randomized controlled trials, cohort studies, case–control studies, ecological studies, or cross-sectional studies regarding the relationship between MNPs and obstetric outcomes, including stillbirth, miscarriage, low birth weight, intrauterine growth restriction (IUGR), or PTB. The exclusion criteria were review studies, studies for which the original manuscript could not be located, and those that did not include any obstetric outcomes. There were no restrictions on species, language, or geographical area.

Disagreements were resolved by discussion and consensus. Data were then collected by reading the full articles that were finally included.

### 2.3. Data Extraction

The following data were extracted from all the studies selected for the review: first and last author details, article publication year, journal where it was published, country of location of the study, study design, species (human or animal) studied, sample size (N), inclusion criteria, plastic detection method, subtype of MNPs, organs in which MNPs are searched, way of administration of MNPs in experimental studies, obstetric outcome studied, results and conclusions.

### 2.4. Assessing the Quality of the Studies and the Risk of Bias

The authors used the Newcastle-Ottawa Scale (NOS) to assess the study quality of the human observational studies [39]. The NOS has three domains: selection of the study population, comparability, and outcomes [39]. Each study is evaluated in each domain and assigned a number of stars based on the adequacy of the task in that domain. In the selection category, the authors primarily assessed the representativeness of the cohort, whether it was exposed or not. Regarding the comparability category, the authors evaluated whether the cohorts used were comparable based on the design or analysis. In the outcome domain, the authors evaluated the assessment of the outcome and the adequacy of the follow-up of the cohorts in terms of the outcome occurring, if that happened [39].

The methodological quality and risk of bias of the included experimental animal studies were assessed using the SYRCLE’s Risk of Bias (RoB) tool, which is specifically designed for preclinical animal research and adapted from the Cochrane Risk of Bias tool [40]. This instrument evaluates potential bias across six domains: selection bias, performance bias, detection bias, attrition bias, reporting bias, and other sources of bias [40].

Two authors independently examined the study quality and assessed the risk of bias in individual studies at both the study and outcome levels. Disagreements were solved by consensus.

## 3. Results

### 3.1. Characteristics of Included Studies—Study Design and Populations

**Figure 1:**
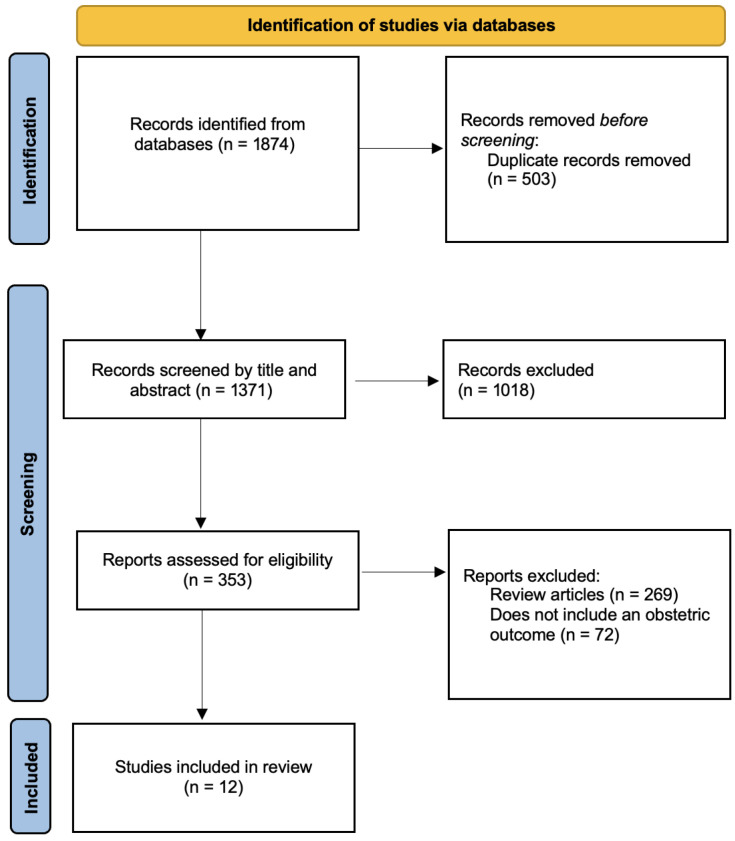
PRISMA: Preferred reporting items for systematic reviews [38].

The search, conducted in accordance with the Section 2, identified 1874 relevant articles in the databases. A total of 12 studies meeting the inclusion criteria were finally included in the review [Figure 1]. The studies were conducted between 2022 and 2025. Most studies were performed in China, followed by Canada, the United States, and Iran [Table 1, Table 2 and Table 3].

The characteristics of the included studies are presented in Table 1, Table 2 and Table 3.

Six studies employed an experimental design [41,42,43,44,45,46], five had an observational design [8,47,48,49,50], and one comprised two different components —an observational and an experimental arm [51].

All experimental studies employ *Muridae* as the model family, given their experimental simplicity and the lower ethical implications [41,42,43,44,45,46,51], with sample sizes ranging from 10 to 82 animals [Table 1], whereas all observational studies involved human subjects [8,47,48,49,50,51], with sample sizes ranging from 10 to 158 participants [Table 2].

In experimental settings, the authors investigated the effects of oral exposure to MNPs, administered through gavage or drinking water to pregnant mice or rats, using a wide range of particle sizes and concentrations [41,42,43,44,45,46,51] [Table 1].

In observational settings, biological matrices analyzed included mainly placental tissue—as it represents the maternal-fetal interface, villous chorionic tissue and amniotic fluid [8,47,48,49,50,51] [Table 2].

To improve clarity and interpretability, results are presented separately for human observational studies and animal experimental studies, given their different levels of clinical applicability [Table 1 and Table 2].

### 3.2. Outcome-Specific Results

#### 3.2.1. Animal Experimental Evidence

Experimental studies in animal models explored the potential biological effects of controlled exposure to MNPs on pregnancy outcomes [Table 1].

Several experimental studies reported associations between MNPs exposure and impaired **fetal growth** [41,42,43,44,45,46]. Aghaei et al. observed a 12% decrease in fetal weight in mice at the highest exposure concentration [45]. Similarly, other studies reported decreased fetal weight, crown–rump length, and fetal growth restriction following MNPs exposure [41]. Additionally, a reduction in daily maternal weight gain during pregnancy has been described in exposed mice [42].

Dose-dependent effects were also reported, with higher exposure levels associated with greater reductions in fetal weight, metabolic disturbances, and skeletal development [44]. Furthermore, alterations in gut microbiota have been proposed as a potential mechanism mediating IUGR in mice exposed to MNPs [41].

A fetus with IUGR is diagnosed based on the combination of fetal weight assessment and Doppler studies, the latter representing fetal hemodynamics [52]. In situations of hypoxia, adaptive mechanisms of blood flow centralization can be identified through Doppler analysis of the umbilical artery and the middle cerebral artery [52]. In this context, Hanrahan et al. demonstrated that MNPs exposure was associated with an increased umbilical artery flow in 43% of exposed mice, suggesting a potential alteration in placental function [43]. However, statistical significance was not reached in the estimated fetal weight or other obstetric outcomes in mice [43]. Another study in rats demonstrates a significant decrease in placental weight in treated dams with MNPs compared to untreated controls. Nonetheless, the authors did not show significant differences in fetal weights. These findings may be influenced by the small sample size (N = 10) [43,46].

Regarding **miscarriage**, experimental evidence suggests that exposure to polystyrene (PS) at 50 or 100 mg/kg may induce miscarriage through activation of apoptotic pathways involving Bcl2/Cleaved-caspase-2/Cleaved-caspase-3 signaling via the mitochondrial pathway [51]. This effect was shown to be partially reversible with Bcl-2 supplementation [51].

Overall, animal studies suggest a potential biological effect of MNPs on obstetric outcomes, although these findings derive from experimental models and are not directly translatable to human clinical settings.

#### 3.2.2. Human Observational Evidence

In human observational studies, several obstetric outcomes were evaluated, including BW, gestational age (GA) at birth, and miscarriage [Table 2].

Regarding **GA at birth** in human subjects, some studies reported an association between higher MNPs exposure and shorter gestational duration [8,47,49]. The largest study, including 159 patients, found that cumulative MNPs levels were 28% higher in preterm placentas compared to term deliveries [47]. This assertion was further supported by another study reporting an inverse relationship between the MNPs concentration in human amniotic fluid and GA at birth [8]. However, one of the studies included in the review lacked a control group and included only human pregnancies complicated by premature rupture of membranes, limiting the interpretability of its findings [49].

Concerning **BW**, several studies identified an inverse association between MNPs exposure and BW [47,50]. Amereh et al. reported a significant negative correlation between placental MNPs levels and BW (r = −0.82), as well as neonatal length (r = −0.56) and head circumference (r = −0.50) in humans, all with *p* < 0.001 [50]. The authors compared the concentration of MNPs in human placentas from 13 IUGR pregnancies with 30 normal pregnancies [50]. To minimize bias, exclusion criteria included patients with other etiologies of IUGR, such as intrauterine infections, high blood pressure, or toxic intake [50]. Similarly, another human study observed an inverse relationship between BW and the concentration of polycarbonate (PC) [47].

However, other studies did not observe statistically significant associations after adjustment for confounding factors [8].

Regarding **miscarriage,** two studies in humans reported a higher presence of MNPs in the villous tissues from pregnancies complicated by miscarriage compared to controls, suggesting a possible association between MNPs exposure and unexplained miscarriage [48,51].

Regarding **other obstetric outcomes**, one study in humans reported that higher levels of terephthalate (PET) and polyurethane (PU) were associated with preeclampsia, while lower levels of MNPs were observed in cases of gestational diabetes [47].

Overall, human studies suggest potential associations between MNPs exposure and adverse obstetric outcomes; however, findings do not allow causal inference.

### 3.3. Type of Polymers Studied

**Figure 2:**
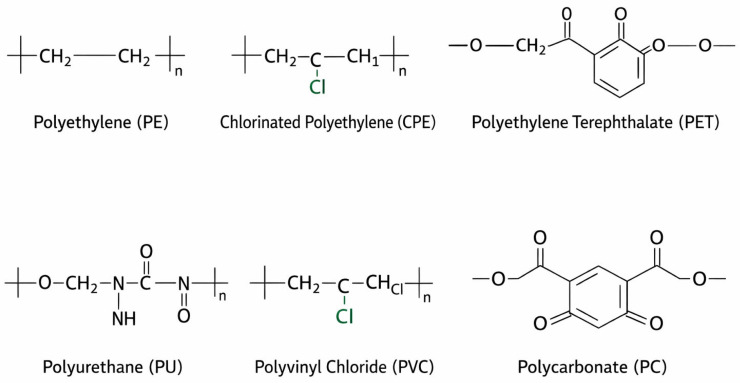
Schematic representation of the main polymers created by the authors [53].

The **most prevalent polymers** detected in amniotic fluid were polyethylene (PE 38.80%) and chlorinated polyethylene (CPE 26.98%) [Figure 2], with 87.56% of the MNPs 20–100 ug in size [8]. Regarding the placenta, the main MNPs detected were PE and PS [50]. Another study described lower particle counts in the amniotic fluid compared to the placenta in the majority of the patients, functioning as a barrier [49].

In the experimental studies, the researchers chose PE to be the MNPs administered to the exposed mice in some studies [43,46] and PS in Aghaei et al. [45].

The study on GA observed different proportions of MNPs, with polyvinyl chloride (PVC), PET, PU, and PC being higher in preterm births. At the same time, acrylonitrile butadiene styrene (ABS) was higher in terms of births [47]. Furthermore, significant inverse correlations were identified between BW and placental PU and PC [47].

Regarding miscarriage, a study described PS as the main MNPs detected [51], but another selected PVC [48].

### 3.4. Polymer Detection Techniques

The most common **techniques for the detection** of MNPs in tissues are Raman microspectroscopy [50], digital microscopy, Fourier Transform Infrared (FTIR) [49], and Pyrolysis gas chromatography-mass spectrometry (Py–GC/MS) [47,51]. Some studies used laser direct infrared spectroscopy (LDIR) to measure MNPs in amniotic fluid from cesarean sections (to avoid contamination of the birth canal) [8]. Another uses a Hyperspectral Microscopy [46]. Each technique has its specific strengths and limitations: **Raman microspectroscopy** allows chemical identification of individual particles with high spatial resolution and is particularly suitable for small particles (<10 µm), including NPs; however, it is time-consuming, susceptible to fluorescence interference, and limited by low throughput [50]. FTIR spectroscopy, especially in its micro-FTIR and imaging modes, enables robust polymer identification and automated analysis, but its spatial resolution restricts reliable detection to particles typically >10–20 µm [49]. Digital or optical microscopy is often used as a preliminary screening tool to assess particle abundance and morphology. Yet, it lacks chemical specificity and may lead to misidentification without spectroscopic confirmation [49]. Py–GC/MS provides highly sensitive and specific polymer identification and quantification, independent of particle size; nevertheless, it is a destructive technique that does not provide information on particle number, size distribution, or morphology [47,51]. In addition, LDIR spectroscopy has been applied for rapid, automated detection of microplastics in biological fluids, offering improved speed and reduced operator bias. However, its applicability to nanoplastics remains limited, and the technique is still under validation for complex tissue matrices [8]. Consequently, the choice of analytical method is largely driven by particle size, matrix complexity, and the specific research question.

**Table 1:** Studies with animals were included in the systematic review.

First Author, Year	Country	Study Design	Population (N)	Plastic Detection Method/Administration	Organ	Obstetric Outcomes Studied
Cary et al., 2023 [46]	USA	Experimental	Rats (10)	Administration of gavages of 10 mL/kg of 250 μg/mL 25 nm carboxylated polystyrene spheres.	Placenta and fetal tissues	Number of fetuses per litter, fetal weight, placental weight, placental efficiency
Bai et al., 2024 [42]	China	Experimental	Mice (30)	Administration of gavages of MNPs at 0, 25, 50, 100 mg/kg body. Confocal and fluorescence microscopy	Placenta and embryos	Weight gain
Hanrahan et al., 2024 [43]	Canada	Experimental	Mice (35)	Administration of 106 ng/L of 740–4990 nm polyethylene with or without surfactant in drinking water	-----------	IUGR
Chen et al., 2023 [44]	China	Experimental	Mice (40)	Administration of 100 nm, 1 and 10 mg/L via drinking water. Confocal microscopy	Placenta and fetal tissues	Fetal weight
He et al., 2025 [41]	China	Experimental	Mice (40)	Administration of gavages of MNPs at 0, 25, 50, 100 mg/kg body weight Fluorescent and 16S sequencing	Placenta and intestines	IUGR
Aghaei et al., 2022 [45]	Canada	Experimental	Mice (82)	Administration of 5 μm or 50 nm polystyrene plastics in filtered drinking water at concentrations of 102, 104 or 106 ng/L	-----------	Fetal weight
Wan et al., 2024 [51]	China	Second part: Experimental	Mice (18)	Administration of pregnant mice with varying doses (0, 25, 50, or 100 mg/kg) of MNPs by oral gavage.	Villous tissue	Miscarriage

Notes: MNPs: Micro and nanoplastics; IUGR: intrauterine growth restriction.

**Table 2:** Studies with humans were included in the systematic review.

First Author, Year	Country	Study Design	Population (N)	Plastic Detection Method/Administration	Organ	Obstetric Outcomes Studied
Wan et al., 2024 [51]	China	First part: Case–control	Human (36)	Transmission electron microscopy and Py-GC/MS	Villous tissue	Miscarriage
Jochum et al., 2025 [47]	USA	Nested cohort	Human (158)	Pyrolysis gas chromatography-mass spectrometry (Py-GC/MS)	Placenta	PTB BW Preeclampsia Gestational diabetes
Wang et al., 2025 [48]	China	Cross-sectional	Human (31)	Raman microspectroscopy and Py-GC/MS	Placental chorionic villi	Miscarriage
Xue et al., 2024 [8]	China	Analytical cross-sectional observational study	Human (40)	Laser direct infrared spectroscopy	Amniotic fluid	GA at birth
Halfar et al., 2023 [49]	China	Observational	Human (10)	Fourier transform infrared spectroscopy	Placenta and amniotic fluid	PTB
Amereh et al., 2022 [50]	Iran	Case–control study	Human (43)	Digital microscopy and Raman microspectroscopy	Placenta	BW Newborn length Head circumference 1 min Apgar score

Notes: Py-GC/MS: Pyrolysis gas chromatography-mass spectrometry; PTB: Preterm birth; GA: gestational age; BW: birth weight; IUGR: intrauterine growth restriction.

### 3.5. Quality Assessment

The methodological quality of the included human observational studies was assessed using the NOS [39] [Table 3]. Overall, six human observational studies were evaluated [8,47,48,49,50,51]. Two studies were rated as high quality (NOS 7–8 stars), supported by stronger population selection, more appropriate comparability, and high-specificity MNPs analytical methods [47,50]. Four studies were classified as moderate quality (NOS 5–6 stars), most commonly due to small sample sizes, limitations intrinsic to cross-sectional designs, and/or incomplete adjustment for key confounders, despite using robust detection techniques [8,48,49,51] [Table 3].

Seven experimental studies conducted in murine models were assessed by the RoB tool [41,42,43,44,45,46,51]. Overall, the studies demonstrated low risk of bias in domains related to outcome data completeness and selective outcome reporting, as all reported outcomes were predefined and fully described. However, an unclear risk of bias was frequently identified in domains related to random sequence generation, allocation concealment, and blinding, as these methodological details were not consistently reported. The high proportion of ‘unclear’ risk of bias judgments reflects insufficient reporting of methodological details rather than confirmed methodological weaknesses [41,42,43,44,45,46,51].

In the study with a double design, including human observational and animal experimental components [51], the animal experimental component was assessed using the RoB tool and the human observational component by the NOS [51].

A meta-analysis was not feasible due to heterogeneity in study design, exposure assessment, and outcome definitions.

**Table 3:** Quality assessment of the studies including humans according to NOS.

Study (First Author, Year)	Study Design	Selection	Comparability	Exposure/Outcome	NOS Score	Quality Level
Jochum et al., 2025 [47]	Nested cohort	★★★★	★★	★★☆	8/9	High
Amereh et al., 2022 [50]	Case–control	★★★☆	★★	★★☆	7/9	High
Wang et al., 2025 [48]	Cross-sectional	★★★☆	★☆	★★☆	6/9	Moderate
Xue et al., 2024 [8]	Analytical cross-sectional	★★★☆	★☆	★★☆	6/9	Moderate
Halfar et al., 2023 [49]	Observational	★★☆☆	★☆	★★☆	5/9	Moderate
Wan et al., 2024 [51] *	Case–control *	★★★☆	★☆	★★★	6/9	Moderate

Notes: * Only the human component of the study was evaluated by NOS.

## 4. Discussion

Since the first detection of MNPs in the placenta, many authors have investigated how these particles manage to cross the maternal-fetal barrier [13]. Although many polymers remain in the placenta, a proportion of them manage to reach amniotic fluid, meconium, and other fetal tissues [49]. The levels of MNPs in the different biological samples vary across studies, likely due to methodological heterogeneity and differences in detection techniques [13,16,49,54].

### 4.1. Summary of Main Findings

This systematic review provides an updated synthesis of the current evidence regarding the potential impact of MNPs on obstetric outcomes. Compared with previously published reviews, which have largely focused on detection and mechanistic hypotheses, this review provides a clinically oriented synthesis of the available evidence on obstetric outcomes, while also incorporating methodological considerations such as detection techniques and polymer variability [17,28,31,32,33]. Overall, the available literature suggests possible associations between MNPs exposure and adverse outcomes such as PTB, low BW, IUGR, and miscarriage. However, the evidence remains limited, heterogeneous, and in some cases inconsistent, precluding definitive conclusions.

### 4.2. Evidence from Animal Experimental Studies: Mechanistic Insights

Experimental studies in animal models provide important mechanistic insights into the potential biological effects of MNPs exposure during pregnancy. These studies have demonstrated associations between MNPs exposure and fetal growth restriction, placental dysfunction, metabolic alterations, and activation of inflammatory and apoptotic pathways [41,42,43,44,45,46]. Dose-dependent effects have been described, with higher exposure levels associated with greater biological impact [44].

However, not all studies report consistent findings, as some experimental models did not demonstrate significant effects on fetal growth or pregnancy outcomes [43,46].

Results from animal studies were interpreted as providing moderate-quality supportive evidence for biological plausibility, rather than definitive causal inference. Although animal studies provide valuable evidence supporting biological plausibility, their findings are not directly translatable to clinical practice.

### 4.3. Evidence from Human Observational Studies: Clinical Perspective

Evidence derived from human observational studies suggests potential associations between MNPs exposure and adverse obstetric outcomes. Higher levels of MNPs have been reported in placentas from preterm births, and inverse relationships with GA and BW have been described in some studies [8,47,49,50]. Additionally, increased abundance of MNPs has been observed in cases of miscarriage and certain pregnancy complications, such as preeclampsia [47,48,51].

Nevertheless, these findings are not consistent across all studies [8,47,50]. Some of the investigations did not identify statistically significant associations after adjustment for confounding factors, highlighting the uncertainty surrounding the clinical relevance of MNPs exposure [8]. Moreover, most studies are limited by small sample sizes, cross-sectional designs, and variability in exposure assessment and analytical techniques. The fetal weight or its evolution to IUGR is the final sign of a complex pathophysiological process that encompasses multiple maternal-fetal mechanisms. Fetuses with greater metabolic reserves may be able to compensate for the harmful effects of MNPs. Therefore, establishing causality with MNPs based on the articles published to date is extremely challenging due to the presence of other potential confounding factors, especially when translating this to human clinical practice.

Consequently, causality cannot be established, and the direct clinical impact of MNPs exposure during pregnancy remains unclear.

### 4.4. Integration of Human and Experimental Evidence

Taken together, the available evidence suggests that animal studies support the biological plausibility of the associations observed in human populations. However, given the limitations of observational human studies and the lack of direct translatability of experimental models, current data remain insufficient to establish a causal relationship between MNPs exposure and adverse obstetric outcomes.

At present, findings should be interpreted with caution, distinguishing between experimental signals and clinically meaningful effects. This distinction is particularly relevant given the physiological differences between species, including body surface area, metabolism, and exposure routes, limiting the direct comparability between murine models and human populations. Furthermore, the doses used in experimental animal studies often exceed those observed in real-life human exposure scenarios. In the studies included in this review, experimental models administered MNPs at doses ranging from 25 to 100 mg/kg/day or concentrations up to 10^6^ ng/L in drinking water [41,42,45]. Using body-surface-area normalization, the highest doses administered in murine studies included in this review correspond to human equivalent doses of approximately 8 mg/kg/day, or around 480 mg/day for a 60 kg adult. In contrast, upper estimates of human microplastic intake have been reported to be around 140 mg/day [55]. These findings suggest that experimental exposures may exceed current estimates of human intake, although direct translational interpretation remains limited by differences in particle characteristics, exposure routes, and bioavailability. Nevertheless, animal models remain essential to establish dose–response relationships and to identify potential toxic effects under controlled conditions.

### 4.5. Mechanisms Underlying MNPs-Induced Damage

The mechanisms underlying MNPs-induced damage have been a primary target of study for researchers in recent years. Impaired placental function has been hypothesized to occur through inflammation, placental apoptosis, and endoplasmic reticulum stress, mediated by the GRP78/IRE1α/JNK axis activation [42]. Furthermore, the potential modulation of epigenetic mechanisms and identification of biomarkers, as well as various parallel miRNA pathways, specifically the KSR-ERK-MAPK pathway, FOXO-Insulin cascade, and GPX3-HIF-α, in humans may be influenced by MNPs exposure [56]. This influence may lead to disruptions in key metabolic and immune pathways, including glucose balance, apoptosis [11], cell proliferation, and angiogenesis [56]. MNPs have the potential to cause metabolic disorders [27] and immune disturbances [57], increasing the percentages of helper T cells while reducing natural killer cells, resulting in an immunosuppressive state [57]. MNPs may even alter the gut microbiota [54], which could lead to adverse obstetric outcomes through placental metabolic disturbances and oxidative stress [41].

Different theories have been proposed to explain the mechanisms underlying the association with a higher miscarriage rate, demonstrating that MNPs activate autophagy and promote the autophagic degradation of SOX2, thereby suppressing SOX2-mediated ROCK1 transcription [51].

### 4.6. Strengths and Limitations

The strengths of this study lie in its extensive literature review, its systematic methodology, adherence to PRISMA guidelines, and the integration of both human and experimental evidence, providing a comprehensive overview of the field. The review followed a predefined protocol with structured search strategies, selection criteria, and risk-of-bias assessment, increasing methodological rigor.

The major limitation of the available evidence is the substantial heterogeneity across studies, including different polymer types, particle sizes, exposure routes, administered doses, analytical methods and biological matrices. This variability complicates comparisons between studies and limits the generalizability of findings.

Furthermore, the review has a relatively small number of included studies (n = 12), which limits the robustness and generalizability of the conclusions. In addition, conclusions are constrained by the quality and reporting of the included studies, particularly regarding exposure assessment and contamination control. An important methodological concern is the potential for other types of contamination. It is indeed recognized that atmospheric pollution, including polycyclic aromatic hydrocarbons, which have also been detected in placental tissue and umbilical cord blood, has been associated with endocrine-disrupting pathways during pregnancy, including documented reductions in β-hCG and progesterone levels [58]. In addition, experimental evidence suggests that exposure to certain environmental contaminants, such as ozone, may increase the sorption capacity of MNPs, indicating a potential interaction between MNPs and co-existing pollutants [59]. Moreover, the diffusion coefficients of different polymers should be considered when assessing the potential for ingestion and biological availability of MNPs across organisms [60]. Contamination control was not uniformly reported across the included studies. While some studies explicitly reported the implementation of contamination control measures—such as the use of procedural blanks, filtered air environments, or non-plastic materials like glass vials [8,47,48,51]—others did not clearly report these strategies [46,49,50]. This heterogeneity in contamination control may affect the reliability and comparability of the reported MNPs levels. Accordingly, the authors acknowledge that the contribution of adsorbed micro-pollutants represents a relevant limitation when interpreting observed physiological and cellular changes attributed to MNPs exposure.

Furthermore, given the ubiquitous presence of MNPs in the environment, secondary contamination may occur during sample collection, handling, processing, or analysis through laboratory materials and equipment. Notably, many of the studies included in this review do not consistently report contamination control measures, which may affect the reliability of the results.

In addition, it should be acknowledged that publication bias may influence the available evidence, as studies reporting non-significant or null associations may be underrepresented in the literature, potentially leading to an overestimation of positive findings.

### 4.7. Implications for Future Research

After reviewing the evidence, the authors conclude it remains scarce, with no standardized metrics or dose–response assessments. Current data confirm the presence of MNPs in the placenta and fetal tissues, suggesting that their accumulation may have deleterious effects on pregnancy outcomes. Nevertheless, higher-quality evidence is required to establish causality. Future studies should aim to standardize methodologies for MNPs detection, exposure assessment, and reporting. Rigorous contamination control protocols should be implemented and transparently described. In addition, well-designed prospective cohort studies with larger sample sizes are needed to better characterize exposure levels during pregnancy and to evaluate potential dose–response relationships.

Experimental studies should aim to use exposure levels that more closely reflect real-world conditions, improving their translational relevance. Integrative approaches combining human and experimental data may help to bridge the gap between mechanistic understanding and clinical implications.

Given the growing relevance of this topic, several ongoing high-quality studies aim to clarify the association between MNPs and adverse obstetric outcomes: UPRISE (*Unraveling ultrafine particulate matter and micro-nano plastics’ mechanisms of impact on fetal health*) [61]; DIMPLE (*Developmental impacts of microplastics exposure in early life*) [62]; and AURORA (*Investigating Exposure and Hazards of Micro- and Nanoplastics During Pregnancy and Early Life*) [63]. Research also focuses on fertility health, such as the *detection of microplastics in human granulosa cells and in the follicular fluid of women undergoing ICSI treatment* [64] and other systemic symptoms, including PLANET (*Exploring the role of plastics and toxins in intestinal inflammation* [65], *biomonitoring of internal exposure to MNPs in patients with chronic kidney disease*) [66], and PECCAD (*Prenatal exposure to emerging contaminants and children’s atopic dermatitis*) [67].

Just as policies aimed at reducing gaseous pollutants have yielded significant health benefits [35], these and forthcoming high-quality studies will shed light on clarifying exposure routes, biological mechanisms, and clinical consequences that will serve as a foundation for the future preventive policy strategies.

## 5. Conclusions

This systematic review provides an updated synthesis of the potential obstetric risks associated with MNPs exposure, drawing attention to its contribution to PTB, low BW, IUGR, and miscarriage. Although the available evidence suggests biologically plausible associations, current data remain limited by heterogeneity in study design, exposure assessment, and analytical methodologies.

Robust scientific evidence—large-scale and well-designed prospective cohort studies—is needed to characterize exposure levels during pregnancy and to clarify dose–response relationships; to assess the potential harm posed by MNPs and to guide the development of effective risk mitigation strategies.

## Data Availability

No new data were created or analyzed in this study. Data sharing is not applicable to this article.

## Supplementary Materials

The following supporting information can be downloaded at: https://www.mdpi.com/article/10.3390/ijerph23050672/s1, File S1: PRISMA Checklist [68].

## Abbreviations

MNPs	Micro- and Nano-plastics
GA	Gestational age
BW	Birth weight
PTB	Preterm birth
Py-GC/MS	Pyrolysis gas chromatography-mass spectrometry
LDIR	Laser direct infrared spectroscopy
FTIR	Fourier transform infrared spectroscopy
IUGR	Intrauterine growth restriction
PET	Polyethylene terephthalate
PE	Polyethylene
CPE	Chlorinated polyethylene
PU	Polyurethane
PC	Polycarbonate
PS	Polystyrene
PVC	Polyvinyl chloride
ABS	Acrylonitrile butadiene styrene
RoB	Risk of Bias
NOS	Newcastle Ottawa Scale
